# Double esophageal duplication cysts, with ectopic gastric mucosa: a case report

**DOI:** 10.1186/1749-8090-8-221

**Published:** 2013-12-01

**Authors:** Zhefeng Zhang, Feng Jin, Hao Wu, Shulian Tan, Zhuang Tian, Youbin Cui

**Affiliations:** 1 Department of Thoracic Surgery, The First Hospital of Jilin University, 71 Xinmin Street, Changchun 130021, China; 2 The First Hospital of Jilin University, Changchun, China; 3 Department of Nephrology, The First Hospital of Jilin University, Changchun, China; 4 Department of Pathology, The First Hospital of Jilin University, Changchun, China

**Keywords:** Esophageal duplication cyst, Ectopic gastric mucosa, Mediastinal cyst, ‘Wait-and-see’ policy

## Abstract

Esophageal duplication cyst (EDC) is a congenital malformation of the posterior primitive foregut, which mainly occurs in the thoracic esophagus. Here, we describe a 3-year-old Han Chinese boy afflicted with intermittent fever of acute onset and dry cough. Thoracic computed tomography revealed an 10 cm × 5.4 cm × 5.8 cm oval-shaped, cyst-like tumor located in the extrapleural space, extending along the right paravertebral gutter and compressing the trachea forward. An additional small-sized, oval-shaped cyst was identified in the posterior mediastinum, between the esophagus and the spinal column, at the T1 level. During open thoracotomy, under general anesthesia, an opaque, thick-walled, esophageal cyst was revealed not to be in communication with the esophageal lumen or the trachea. This cyst was subsequently resected in an *en bloc* manner. The small (1-cm) esophageal cyst was left untreated based on a “wait-and-see” policy. Histological analysis showed that the resected cyst was walled by hyperplastic, fibrous tissues and locally lined with gastric mucosa inherent glands. This finding was consistent with a diagnosis of EDC, with ectopic gastric mucosa. The respiratory tract symptoms resolved immediately after the operation. Computed tomography obtained at the 6-month follow-up showed that no disease, residual or recurrence, was present after the resection of the large-sized cyst, and the small-sized cyst remained unchanged in size.

## Background

Gastrointestinal (GI) tract duplication, or GI duplication cyst, is a rare congenital malformation of the GI tract. The etiology of GI tract duplication is not fully understood; however, an initial developmental abnormality during the gastrulation stage is thought to be the most likely cause [1]. Based on autopsy reports, it is estimated that GI tract duplication affects 1 in every 4,500 individuals. It predominantly occurs during childhood [2], exhibiting a highly variable clinical presentation, depending on its location and size. Exploratory imaging studies may help identify the duplication cyst in some cases; however, these cannot differentiate its pathology from Meckel diverticulum or other GI cyst-like diseases [3]. Therefore, definitively diagnosing GI tract duplication remains a challenge to clinicians.

GI tract duplication mainly occurs in the ileum of the small intestine, which accounts for over 40% of cases [2]. Esophageal duplication cysts (EDCs) are much rarer, comprising 4% of all cases and 10-15% of all foregut duplication cysts [4]. EDC was initially characterized by Blasius in 1711 [5] and was previously categorized as a type of esophageal cyst, due to the duplication of the submucosal and muscular portions of the esophagus. EDC may communicate with the native lumen of the esophagus. EDC is also the second most common benign posterior mediastinal lesion in children, following bronchogenic cyst. The incidence rate of EDC is 1 in 8,200, with a two-fold male prevalence over women [4]. While EDC may occur in the cervical [6] and abdominal segments [7] of the esophagus, it mainly occurs in the thoracic segment [8]. The pathogenic mechanisms of EDC are unknown; however, it is thought to be associated with abnormal esophageal development occurring in the fifth to eighth week of gestation, when the posterior primitive foregut coalesces to form a single esophageal lumen. EDC is relatively common in children presenting with mediastinal masses, accounting for 30% of all pediatric posterior mediastinal masses [4].

Esophageal duplication cysts may contain ectopic gastric mucosa [9] and pancreatic components [10]. Ectopic GI mucosa of the esophagus is thought to result from the reduced replacement of simple column epithelium by stratified squamous epithelium during esophageal embryogenesis. This condition is usually incidentally detected by endoscopy or biopsy and manifests at the gastric mucosal inlet. Ectopic GI mucosa can complicate the symptoms of GI tract duplication, especially those involving the ileum, by mimicking Meckel diverticulum [3]. Patients may display symptoms of gastrointestinal bleeding, obstruction, diverticulitis, and umbilical abnormalities. However, few studies in the literature have addressed EDC with ectopic gastric mucosa.

In this case study, we described a boy afflicted with intermittent fever of acute onset and dry cough, and diagnosed as having double EDCs containing ectopic gastric mucosa. The larger cyst was resected *via* thoracotomy, whereas the smaller cyst was left untreated, based on a “wait-and-see” policy.

## Case presentation

A previously healthy 3-year-old Han Chinese boy, who presented with intermittent fever of acute onset and dry cough, but without dysphagia, was referred to our Department of Pediatric Respiratory Medicine. He showed no clinically significant abnormalities on physical examination, except for feebleness and pyrexia (39.0°C). Chest radiography and computed tomography (CT) scans at the initial visit revealed a mediastinal tumor with complicating pneumonia. He was subsequently referred to our pediatric thoracic surgery service.

**Figure 1:**
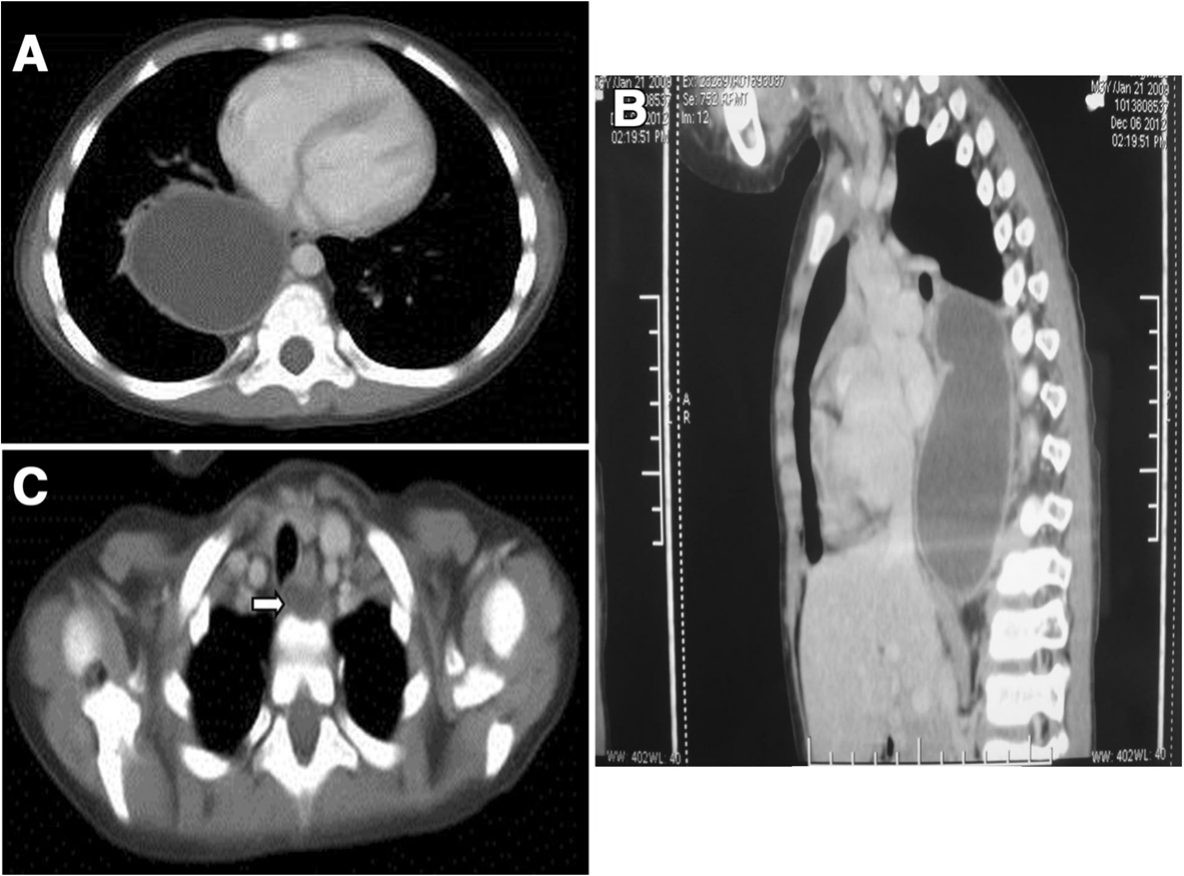
**Computed tomography scan of esophageal duplication cysts. (A)** Transverse and **(B)** sagittal views of the large-sized cyst; and **(C)** transverse view of the small-sized cyst (white arrow).

**Figure 2:**
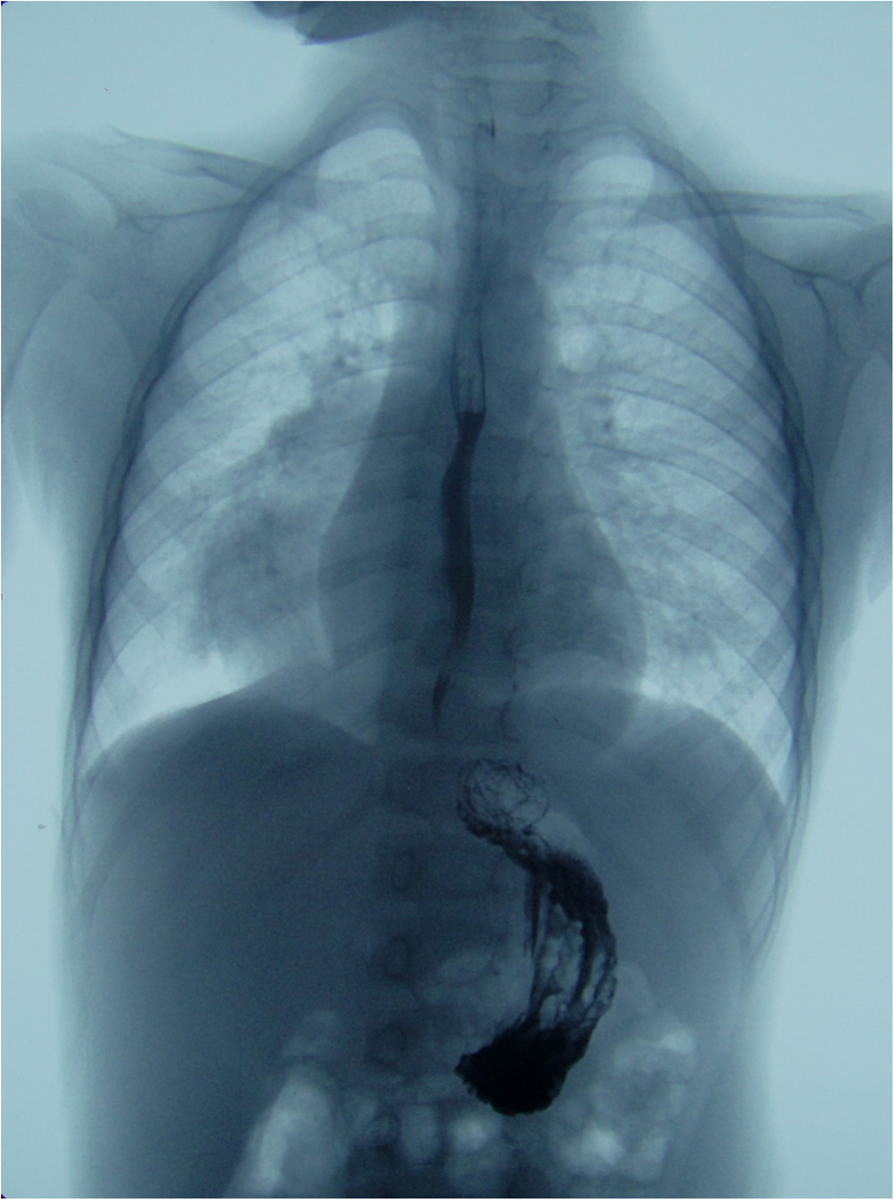
Esophagography excluded the possibility of esophageal stenosis, atresia, or tracheoesophageal fistula.

Three-dimensional CT reconstruction demonstrated that two oval-shaped cystic masses were located in the posterior mediastinum, which displayed well-defined margins and were fluid-filled, yet free of any air-fluid levels. The larger cyst was 10 cm × 5.4 cm × 5.8 cm in size. It was attached to the middle and lower portions of the esophagus in the extrapleural space and extended along the right paravertebral gutter. The cyst compressed the forelying trachea, the right main bronchus, and the right inferior lobe anteriorly (Figure 1A, B). The smaller cyst was located between the posterior wall of the lower cervical esophagus, and the spinal column (level T1), at a size of 1.0 cm × 0.8 cm (Figure 1C). Bilateral bronchopneumonia was evident on chest radiography and CT scan, however, no lymph node enlargements were observed. Contrast-enhanced CT did not reveal any obvious enhancement of either cyst. Diatrizoate meglumine and diatrizoate sodium esophagography excluded the possibility of esophageal stenosis, atresia, or tracheoesophageal fistula (Figure 2). Therefore, the patient was diagnosed with esophageal cyst with complicating bronchopneumonia.

**Figure 3:**
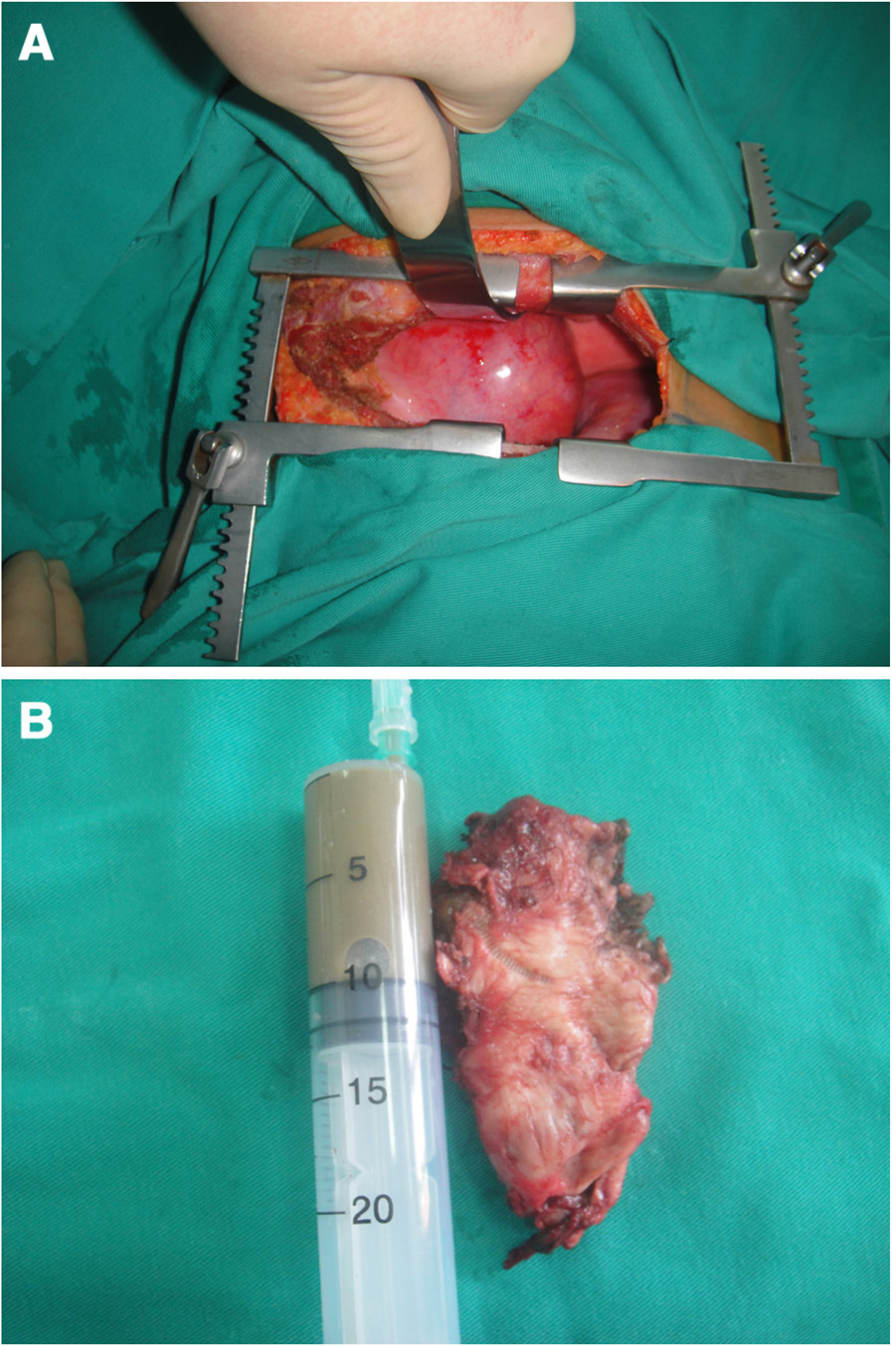
**Intraoperative findings of the large-sized esophageal duplication cyst. (A)** A cystic mass was located in the right-sided posterior mediastinum; and **(B)** gross pathology of the resection specimen containing brownish cystic fluid.

Because the cysts were symptomatic and one of them was oversized, elective open thoracotomy was undertaken. A double-lumen endotracheal tube was used to deflate the right lung, and a right-sided thoracotomy was made along the sixth intercostal space. A 10 cm × 5.4 cm × 5.8 cm cyst originating from the right lateral wall of the esophagus at the level of the carina was identified (Figure 3). The cyst wall was approximately 0.3 cm in thickness and moderately adherent to the membranous part of the right main bronchus and the dorsal segment of the right lower lobe; however, it was not in communication with the esophageal lumen or the bronchopulmonary tree. The cyst was punctured and deflated using an 18-gauge needle. Roughly 200 ml of brownish, viscous fluid, suggestive of intracystic hemorrhage, was drained away. The thoracic duct, esophageal mucosa, and vagus nerve were all well-preserved, and the cyst was mobilized and removed in an *en bloc* manner. The small esophageal cyst was left untreated based on a “wait-and-see” policy. This cyst was located at the thoracic entrance (the junction between the cervical portion and the thoracic portion of the esophagus) and was not easily accessible through the sixth intercostal space incision.

**Figure 4:**
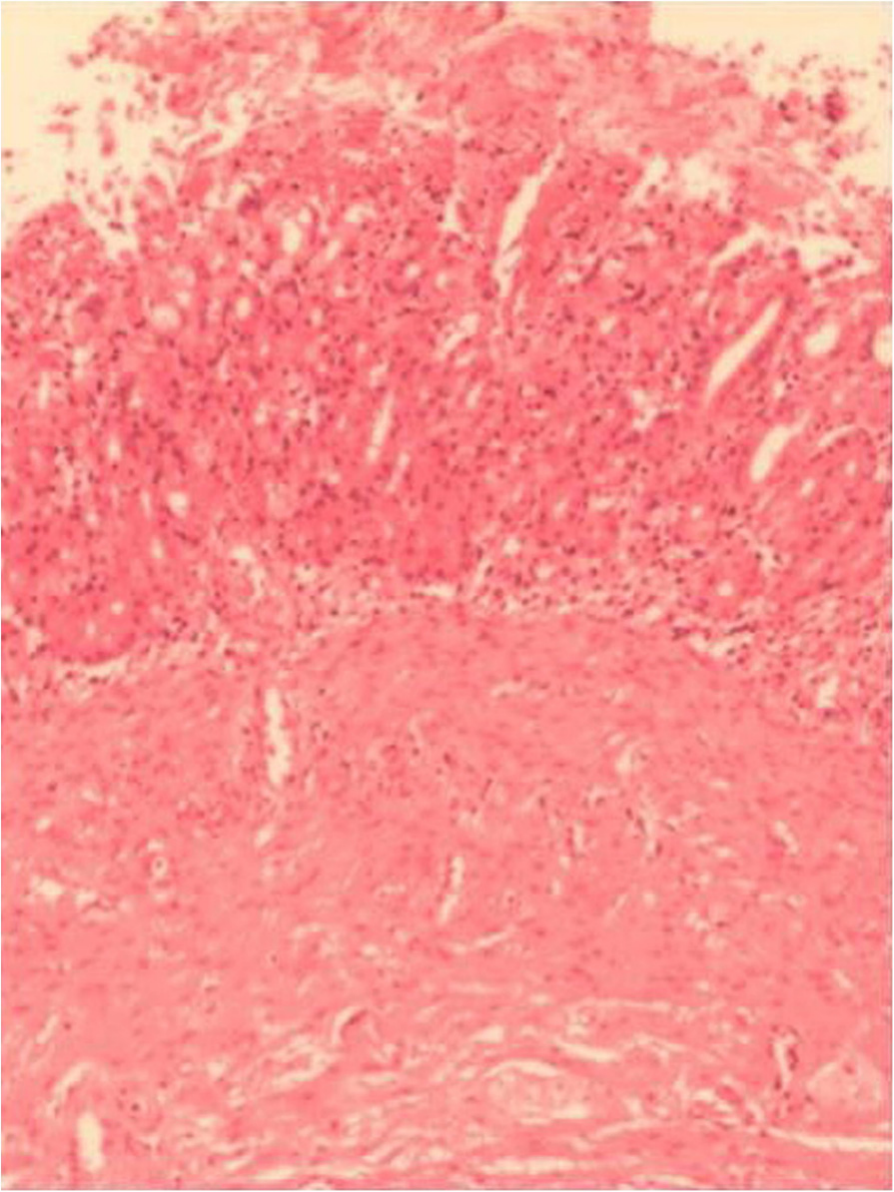
**Pathology of the resected large-sized esophageal duplication cyst.** Histology revealed esophageal duplication cyst with ectopic gastric mucosa (hematoxylin-eosin, 400×).

Gross pathology of the resected specimen showed a grayish cystic mass, with a 0.3-cm-thick wall, lined with brownish, rough epithelial-like tissue. Histological analysis of the resected esophageal cyst revealed that the cyst wall was composed of hyperplastic fibers and lined with stratified squamous epithelium, containing gastric mucosal simple columnar epithelium (Figure 4). The disease was then histologically diagnosed as EDC with ectopic gastric mucosa.

**Figure 5:**
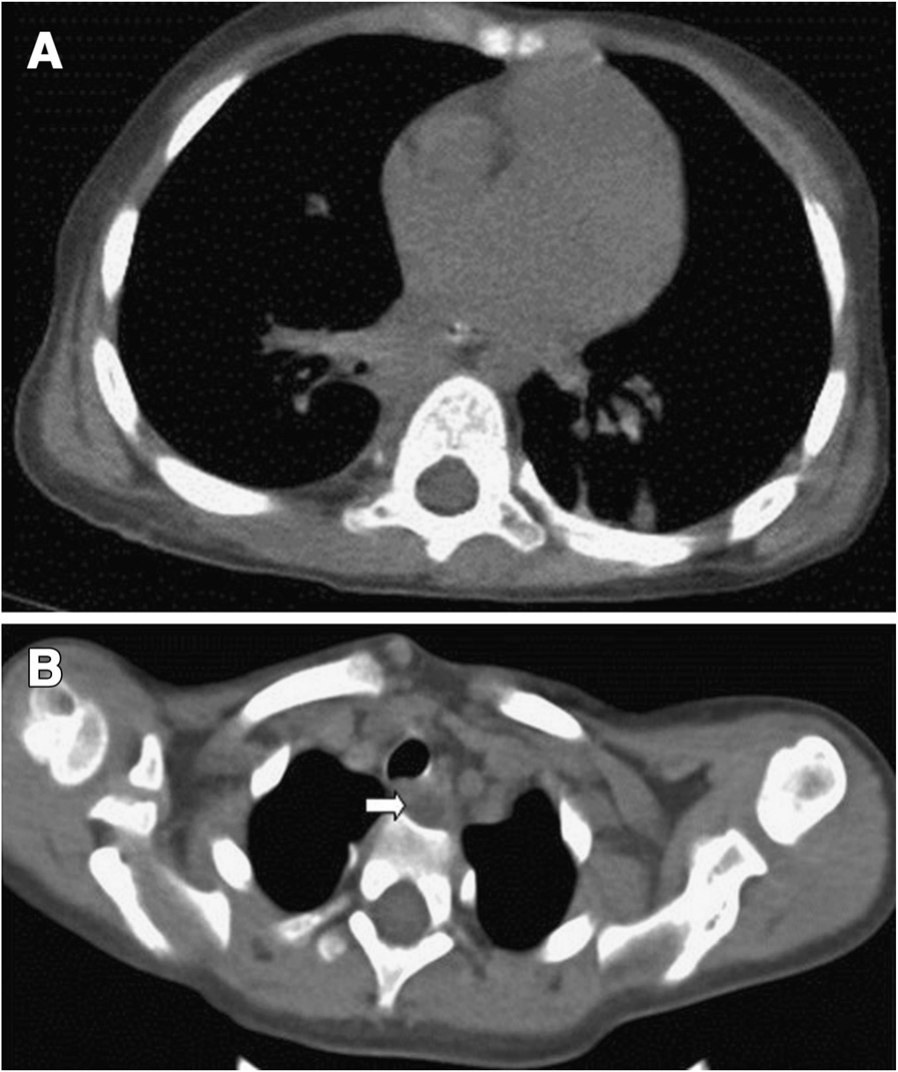
**Follow-up chest computed tomography scan.** No disease, residual or recurrent, was found for the resected large-sized cyst **(A)** and no growth of the small-sized cyst was observed **(B)** (white arrow).

The respiratory tract symptoms resolved immediately after operation. The patient underwent an uneventful recovery. A chest CT scan revealed no residue of the resected cyst on postoperative day (POD) 5. The patient was discharged from hospital on POD 7, and treatment was continued at the outpatient clinic. At the follow-up visit at 6 months, the patient appeared asymptomatic and generally in good health. A follow-up chest CT scan showed that no disease, residual or recurrent, was present after the resection of the large-sized cyst, and the small-sized cyst remained unchanged in dimension (Figure 5).

## Discussion

EDC is the second most common GI duplication cyst, after ileal duplication cyst. Nevertheless, fewer than 100 cases of EDC are currently documented. EDC usually occurs in isolation; however, two cases of multiple (two) EDCs have been reported [11,12]. The present patient appears to be the third case of multiple EDCs ever to be reported.

Most EDCs do not present with debilitating symptoms. Affected children may complain of nonspecific esophagus-related or respiratory tract symptoms, depending on the size and the presence or absence of complicating hemorrhage, infection, rupture, or respiratory tract involvement [13]. EDCs may be asymptomatic and incidentally found thoracotomy for other surgical conditions. Our patient exhibited obstructive pneumonia-associated symptoms caused by the large-sized EDC compression on the tracheobronchial tree, which were not different from those caused by common pediatric mediastinal tumors.

Radiographic manifestations of EDC are generally nonspecific and similar to those of other common mediastinal non-neoplastic or neoplastic diseases. Non-contrast and contrast-enhanced CT scan can demonstrate esophageal cysts and the neighboring esophagus and tracheobronchial tree [14]. If the CT scan produces an equivocal result, a magnetic resonance imaging (MRI) scan [15] and transesophageal endoscopic ultrasonography (EUS) [16] can be used for the preoperative assessment and prenatal diagnosis of EDC [17,18]. A T1-weighted MRI scan can accurately visualize an esophageal cyst, even in the presence of complicating bleeding and infection [19]. EUS is considered to be an effective adjuvant to the radiographic and MR evaluation of EDC, and often shows EDCs as anechoic or hypoechoic cysts [16]. EUS-guided fine needle aspiration biopsy can offer a pathology-based diagnosis, differentiating EDC from other mediastinal tumors [20]. EDCs may occur concomitantly with esophageal stenosis/atresia [21], tracheoesophageal fistula [22], and other tracheoesophageal/pulmonary malformations [23]. Preoperative esophagography and EUS can help exclude coexisting malformations and outline the operative plan. In our patient, chest radiography and CT scan clearly revealed the two EDCs and tracheal involvement, whereas esophagography excluded the possibility of concomitant tracheobronchial malformations.

Histology offers the best diagnostic tool of EDC. The pathological criteria of EDC are as follows [5]: the cyst is attached to the esophageal wall, is communicating with the epithelia originating from the GI tract, and is underlain by two layers of muscularis propria. EDC can be lined by pseudostratified columnar epithelium, gastric mucosal epithelium, and squamous epithelium. The cyst wall may also contain cartilaginous tissue. Ectopic GI mucosa can occur independently of or concurrently with EDC, in the upper and/or middle parts of the esophagus. Presence of gastric mucosa in the lower part of the esophagus is more commonly known as Barrett esophagus [24]. These ectopic gastric mucosae normally manifest as small patches of columnar epithelium containing gastric glands, which vary in size from millimeters to centimeters on endoscopy and are detectable by technetium-99 m pertechnetate scan [3]. Only one case of EDC with ectopic gastric mucosa in an infant presenting with hemoptysis has been reported [14]; respiratory tract bleeding in this patient may have resulted from the corrosive effect of gastric acid secreted by the ectopic gastric mucosa. Ectopic pancreatic tissue was also reported to occur concurrently with EDC [10]. Similar to native gastric mucosa, ectopic counterparts are still subject to the risk of malignant transformation [25].

Surgical excision is the mainstay treatment for symptomatic EDC. This procedure can be performed *via* thoracotomy or thoracoscopy for removing thoracic EDC. Complete removal of EDC is required to minimize the risk of disease recurrence. Thoracoscopic resection is believed to be as effective as the conventional open procedure, and offers additional minimal invasiveness and expedited postoperative recovery [4]. In our patient, the cyst was relatively large (~10 cm) and there was a complicating infection; therefore, thoracotomy rather than thoracoscopy was used to resect the large-sized EDC. Complete [26] or partial endoscopic resection [27] after EUS has also been reported to be an effective and safe treatment for small-sized, pedunculated, and superficially layered EDCs. The small-sized, asymptomatic EDC in our patient was left untreated, based on a “wait-and-see” policy for the treatment of small-sized benign GI cysts that cannot be accessed though a single thoracic incision. A previous study showed that an EDC associated with mild upper GI symptoms remained unchanged on EUS for over 13 years [28]. If necessary, small-sized EDC can be ablated using endoscopic or percutaneous anhydrous ethanol injection [29].

## Conclusions

EDC is an uncommon differential diagnosis in children presenting with posterior-mediastinal cystic masses. The use of a combination of multiple medical imaging techniques, such as esophagography, CT scan, MRI, and EUS, can help characterize EDC in relation to its neighboring anatomy and exclude possible concomitant tracheoesophageal malformations. Ectopic gastric mucosa can be retrospectively detected in EDC resection specimens. Large-sized and/or symptomatic EDCs should be surgically treated, while small-sized and/or asymptomatic EDCs should be closely followed up, especially if the cyst is located in proximity to the superior thoracic aperture or tracheal carina.

## Consent

Written informed consent was obtained from the patient’s guardian for the publication of this case report and any accompanying images. A copy of the written consent is available from the Editor-in-Chief of this journal.

## Abbreviations

CT: Computed tomography; EDC: Esophageal duplication cyst; EUS: Endoscopic ultrasonography; GI: Gastrointestinal; MRI: Magnetic resonance imaging; POD: Postoperative day.

## Competing interests

The authors declare that they have no competing interests.

## Authors’ contributions

YBC, study concept and design; ZFZ and FJ, drafting and finalization of the manuscript, preparation of the figures, acquisition of data, and analysis and interpretation of data; HW and SLT, critical revision of the manuscript for important intellectual content and material support; ZT, preparation of the figures, and technical and material support. All authors read and approved the final manuscript.

## Authors’ information

Dr. Youbin Cui, Superintendent of the Institution, surgeon; Zhefeng Zhang, master’s degree, surgical trainee; Feng Jin, master’s degree, research student; Hao Wu, master’s degree, research student; Shulian Tan, master’s degree, research student; Zhuang Tian, master’s degree, technician.
